# Estimation of Brain Functional Connectivity in Patients with Mild Cognitive Impairment

**DOI:** 10.3390/brainsci9120350

**Published:** 2019-11-30

**Authors:** Laia Farràs-Permanyer, Núria Mancho-Fora, Marc Montalà-Flaquer, Esteve Gudayol-Ferré, Geisa Bearitz Gallardo-Moreno, Daniel Zarabozo-Hurtado, Erwin Villuendas-González, Maribel Peró-Cebollero, Joan Guàrdia-Olmos

**Affiliations:** 1 Departament de Psicologia Social i Psicologia Quantitativa, Facultat de Psicologia, Universitat de Barcelona, 08035 Barcelona, Spain; nuria.mancho.fora@gmail.com (N.M.-F.); mmontala@ub.edu (M.M.-F.); mpero@ub.edu (M.P.-C.); 2 UBICS Institute of Complex Systems & UB Institute of Neurosciences, 08035 Barcelona, Spain; 3 Facultad de Psicología, Universidad Michoacana de San Nicolás Hidalgo, Morelia 58000, Mexico; egudayol14@yahoo.com.mx (E.G.-F.); erwinvilluendas@gmail.com (E.V.-G.); 4 Instituto de Neurociencias, Guadalajara, Jalisco 44130, Mexico; geisabearitz@hotmail.com; 5 CINDFA-Grupo Río, Guadalajara, Jalisco 44648, Mexico; daniel.zarabozo@gmail.com

**Keywords:** fMRI, mild cognitive impairment, aging, functional connectivity

## Abstract

Mild cognitive impairment is defined as greater cognitive decline than expected for a person at a particular age and is sometimes considered a stage between healthy aging and Alzheimer’s disease or other dementia syndromes. It is known that functional connectivity patterns change in people with this diagnosis. We studied functional connectivity patterns and functional segregation in a resting-state fMRI paradigm comparing 10 MCI patients and 10 healthy controls matched by education level, age and sex. Ninety ROIs from the automated anatomical labeling (AAL) atlas were selected for functional connectivity analysis. A correlation matrix was created for each group, and a third matrix with the correlation coefficient differences between the two matrices was created. Functional segregation was analyzed with the 3-cycle method, which is novel in studies of this topic. Finally, cluster analyses were also performed. Our results showed that the two correlation matrices were visually similar but had many differences related to different cognitive functions. Differences were especially apparent in the anterior default mode network (DMN), while the visual resting-state network (RSN) showed no differences between groups. Differences in connectivity patterns in the anterior DMN should be studied more extensively to fully understand its role in the differentiation of healthy aging and an MCI diagnosis.

## 1. Introduction

Mild cognitive impairment (MCI) is a syndrome in which patients have greater cognitive decline than would be expected from normal aging, but not enough to be considered dementia or another disease [1,2]. MCI individuals have relatively preserved daily functioning but cognitive deficits that must be objectively assessed. MCI has also been considered a stage prior to Alzheimer’s disease (AD) or other dementia syndromes [3,4,5] because these patients have an increased probability for a future conversion to those diseases compared to that in healthy controls.

MCI patients also form a remarkably heterogeneous group due to the diverse symptomatology that they can present, and they can also show different levels of deterioration [4,6,7,8].

Several research groups have studied this syndrome to better understand and define an MCI profile, and there have been attempts to find biomarkers for an optimal differentiation between healthy and MCI individuals [9,10] or to predict which patients will convert to AD or dementia [11,12,13]. Some of these studies used functional MRI (fMRI) because it is a technique that provides information about brain activation patterns by assessing blood oxygen level–dependent (BOLD) changes. fMRI is a noninvasive tool that could be used in task paradigms and in resting-state paradigms.

The study of single-vessel fMRI in the characterization of BOLD responses is a very interesting application of fMRI studies. This perspective permits the study of functional connectivity from a vascular perspective, as many authors recently showed [14,15]. For example, [15] found that this approach can help researchers understand individual vascular coupling events in the neuron-glia-vessel network, both in normal and diseased brain states. Additionally, [14] demonstrated the feasibility of applying a multimodal fMRI platform to measure neuronal correlates of resting-state hemodynamic signal fluctuations in humans and rats. Therefore, the vascular perspective entails a very interesting approximation in fMRI studies, although in the present study, it will not be fully discussed.

Many findings have been reported in fMRI studies with MCI patients. One of the most important results has been the verification of the existence of compensatory mechanisms depending on the brain area. Increased activity in particular brain regions in MCI individuals compared to healthy adults has been found, and this increased activity could compensate for deficits in other areas [16,17,18,19]. In addition, the increased activation in the hippocampus when the participant was engaged in memory tasks could predict the early detection of AD; this is a concrete example of compensatory behavior that, in this particular case, was related to prediction techniques of future dementia [20] but could also be related to the need of the brain to use additional resources to confront a particular situation [21]. The role of the hippocampus has seemed to be particularly relevant in this disease [22], but there are not enough studies to determine what features would firmly lead us to a conclusion regarding which people with MCI will convert to AD and which people will not.

The comparison of brain functional connectivity between healthy older adults and MCI patients has also been studied. In addition to compensatory mechanisms, individuals with MCI showed decreased brain connectivity in some regions compared to that in healthy persons. This lower functional connectivity has been found in many regions, such as the hippocampus and other temporal areas [17,23,24,25,26]. Additionally, the default mode network (DMN) showed this decreased function [27,28,29,30,31], but the functions of other resting-state networks (RSNs), as well as the relation between them in terms of the number of connections and the intensity of these connections, have been shown to be unaltered [32,33].

Brain functional connectivity in healthy older adults also has particular characteristics. In comparison to younger adults, older individuals have shown increased internetwork connections and decreased intranetwork connections [34]. The DMN is usually affected by aging, as are other resting-state networks (RSN) [35,36]. Decreased activity appeared to result from a diminution in the number of connections as well as a diminution in the intensity of these connections in many brain regions and networks [37]. These aging-related neural changes could be initiated early in adults, such as in middle age, and accumulate over time [38]. A review of brain functional connectivity changes related to aging can be found in [39].

Although several studies have examined this comparison between healthy adults and those with MCI, it remains difficult to establish an actual profile of MCI individuals that would highlight the differences between the groups in terms of connectivity [40]. The reduction of classification errors is critical to establish any robust comparison between the groups using either classic tests or brain signals [41]. Although the diagnostic criteria are clearly defined and known [42], the selection of a clean sample of false positives has remained a challenge for professionals. The need for many neuropsychological tests, as well as interviews with both the patient and their relatives or caregivers, often makes it difficult to affirm with certainty that a well-diagnosed MCI sample is available [43]. Obviously, this question becomes somewhat more complex if we add the need to exclude other pathologies with similar positive or negative symptoms.

Furthermore, brain connectivity differences in these populations have been noticed but not perfectly characterized, and additional studies of the brain connectivity characteristics are needed. This characterization is usually complicated because of the great difficulty involved in the correct selection of the sample, which can sometimes have a considerable number of participants, but they have not been subjected to a sufficiently exhaustive evaluation. Therefore, additional studies with accurately selected samples are necessary to provide dramatic improvements in our current knowledge about the brain connectivity characteristics in people with this diagnosis.

For these reasons, the main objective of this work is to realize an exhaustive descriptive study to identify brain connectivity patterns that can be identified in a sample of MCI patients and in an age-matched sample of healthy people.

In contrast to similar previous studies, a very thorough selection of participants will be carried out to confirm that the entire sample presents the diagnosis of MCI and thus guarantee the absence of false positives. In addition, analysis techniques that are not very frequently used in this area will be applied in the study of brain connectivity as well as the more usual approaches, so that the potential of these analytic tools can be demonstrated. For this purpose, we propose to use a segregation process of connectivity networks based on the identification of areas of high connectivity density and to analyze their distribution and their relationship with external criteria. Obviously, the most basic comparison will be to analyze whether that segregation is different between MCI subjects and healthy controls. 

Finally, we hypothesize that their brain connectivity characteristics will be different, in concordance with previous literature. In particular, we expect that functional connectivity between resting-state networks will be different between the two groups, with a different number of connections and a different relationship between networks. We hypothesize that most of the correlation coefficients will not be significantly different between groups, knowing that differences between MCI and controls are usually subtle. In addition, and in accordance with previous studies, the analysis of functional connectivity networks will allow the identification of complex behavior patterns that can be interpreted as a reflection of compensatory mechanisms associated with the characteristics of the diagnosed pathology. For example, a study of the patterns of connectivity networks has found that the pattern of complexity in the effective connectivity network was much denser in patients diagnosed with type I diabetes than in a group of healthy people matched by age and years of formal education [44]. This type of statistical approach should show its effectiveness in identifying different patterns of connectivity between subjects diagnosed with MCI and their healthy controls.

## 2. Materials and Methods

### 2.1. Participants

Five hundred thirty possible participants were contacted during the screening phase from January 2017 to October 2018 in the primary care clinical setting. The inclusion criteria were age between 65 and 80 years and having fulfilled the Petersen criteria for an MCI diagnosis. The exclusion criteria included illiteracy or an inability to understand the protocol or undergo neuropsychological tests; any relevant psychiatric illness, advanced cognitive deterioration, dementia or other neurodegenerative diseases (other than MCI); any prior cerebrovascular accident, traumatic brain injury, abuse of alcohol or other substances; and any MRI-related incompatibility (the presence of metallic objects within the body, pacemaker or claustrophobia).

One hundred one of these potential participants met the inclusion criteria and were eligible for further neuropsychological assessment to be potentially included in the MCI group. Of these, 26 refused to participate. Of the 81 remaining patients, 6 were excluded due to fMRI-related incompatibility. Fifteen were excluded due to significant depressive symptoms or alcohol abuse. Others were excluded due to physical diseases that were susceptible to interference with the fMRI data (type 2 diabetes, *n* = 21; hypertension, *n* = 6; history of known or suspected brain injury, including head concussions, *n* = 5; pulmonary and/or cardiac illness, *n* = 6; and type 2 diabetes comorbid with other pathologies, *n* = 8). Thus, 14 patients were included in the study. In relation to the control group recruitment procedure, when an MCI participant was included in the study, a control participant was identified and matched by age and formal education years.

After the scan data were collected, individuals with severe movement artifacts or incomplete rs-fMRI time series were excluded from the analysis. More specifically, data from three individuals in the control group and four in the MCI group were discarded because the absolute root mean square movement was greater than half a voxel [45]. Therefore, the remaining sample had 20 participants (10 MCI and 10 matched controls by age and formal education).

This was a small sample. It was not exceptional, since works in this area have had samples ranging from very large (*n* = 115 MCI [46]) to smaller than the one here (*n* = 9 in [4]). A possible advance, in our opinion an interesting one, is the use of strictly paired samples based on the key variables for the study of cognitive functioning, such as age and years of education. This strategy makes sampling very difficult but guarantees a reduction in the error and residual variance that the sample size does not allow. This reduces, in part, the negative effect of the limited sample size. On the other hand, the sample selection criteria were so rigid in terms of movement and noise in the fMRI register that motion correction was almost unnecessary. Most works with this type of clinical sample are not as rigid in the application of these criteria for noise reduction in the BOLD signal.

Written informed consent was obtained from every individual prior to taking part in the study, according to the Declaration of Helsinki and the institutional ethics committee. Moreover, this procedure was approved by the Bioethical Committee of the University of Barcelona (03/10/2017).

### 2.2. Material

In relation to the neuropsychological assessment of the participants in this study, MCI cognitive functions were assessed in every individual in the MCI and control groups. This neuropsychological evaluation was quite exhaustive and included the administration of the Mini-Mental State Examination (MMSE), Prospective and Retrospective Memory Questionnaire (PRMQ), Geriatric Depression Rating (GDR) scale, Pfeffer’s Functional Activities Questionnaire (PAQ), Clinical Dementia Rating (CDR) scale, Boston Naming Test (BNT) and NEUROPSI Attention and Memory battery. This last test is a brief neuropsychological battery with 28 subtests that has been developed to evaluate the neuropsychological performance in the cognitive domains of orientation, attention and concentration, executive functions, working memory and immediate and delayed verbal and visual memory in psychiatric and neurological patients. Norms adjusted to age and education level are available for the population included in our study [47].

Neuropsychological assessment was divided into two stages that were performed in two sessions at different times (see Procedure section). The first stage included the MMSE and PRMQ. In this phase, the PAQ was also administered to a family member close to the patient when one was available at that time. The second stage included, in this order, the CDR from an informer (a familiar or close person who lived with the participant), BNT, NEUROPSI Attention and Memory and GDR. Additionally, during this assessment, the evaluator asked for other possible exclusion reasons, which were described above, through a semistructured interview specifically designed for the study, and a complete medical and psychological history was provided during the process. They also completed a brief questionnaire to determine the presence of any MRI-related incompatibility.

The MMSE is a 30-item screening tool to evaluate orientations to place and time, immediate and delayed recall, attention and calculation, and language and visual construction [48,49]. The MMSE is used in most studies related to memory, cognitive skills, impairment and aging. The Spanish version proposed and validated by [50] was used, taking into account the adjusted values proposed by [51] in their study of the MMSE in a Mexican population. The internal reliability of the scale was more than acceptable, with an intraclass correlation coefficient of r = 0.88 (*p* < 0.001), while the correlation coefficient in cross-validation was *r* = 0.92.

The PRMQ is a questionnaire of sixteen items concerning memory slips in everyday life [52], including prospective and retrospective memory failures. The Spanish version of this questionnaire was adapted for the Mexican population, and this version had a good internal consistency coefficient (α = 0.89) and adequate test-retest reliability of the total scale and the two subscales (*r* = 0.81, *r* = 0.78 and *r* = 0.80, respectively) [53]. To assess orientation, attention and concentration, executive functions, working memory and immediate and delayed verbal and visual memory but also attention, we used these subscales of the NEUROPSI, a brief neuropsychological test battery with different norms depending on the age and educational level of the participant (Ostrosky-Solís et al., 2007). Test-retest reliability values were acceptable for the total NEUROPSI score (*r* = 0.89), with values between 0.79 and 1 in all the subscales.

The CDR [54,55] and PAQ [56] were used to confirm good functioning in the daily activities of the elderly population, with information provided by a relative or someone close to the participant who lived with them. The CDR is available in many languages, with a reliability of α = 0.83 among investigators in a multicenter study [54], while the PAQ has presented a high interrater reliability among neurologists (*r* = 0.97). The absence of depression was determined by the GDS [57], a common scale used in this population with an internal consistency reliability of α = 0.82 [58]. Finally, language ability and verbal scores were assessed with the BNT [59], which has adequate construct validity in Spanish-speaking populations [60].

### 2.3. Procedure

Neuropsychological assessment was divided into two stages that were performed in two sessions at different times. The first stage was the screening phase and included the MMSE and PRMQ. The PAQ was also administered when there was a relative of the participant available at the time. If the individuals assessed in the screening were able to participate in the study after this phase and did not meet any exclusion criteria, they went through the neuropsychological evaluation phase. This stage included, in this order, the CDR from an informer (a relative or someone close who lived with the participant), the PAQ if it was not applied during the screening, followed by the BNT, NEUROPSI (Attention and Memory) and GDR. Additionally, during this assessment, the evaluator asked for other possible exclusion reasons, which were described above using a semistructured interview specifically designed for the study, and a complete medical and psychological clinical history was obtained during the process. They also completed a brief questionnaire to determine MRI-related incompatibility (claustrophobia, the presence of metal in the body or other circumstances). After the classic evaluation phase, the subjects were summoned for the resonance session a short period of time after the completion of the psychometric record and in accordance with their schedules and availability.

### 2.4. MR Image Acquisition

All participants were scanned with a Philips Ingenia 3.0-T system at the Laboratorio Clínico, Centro Integral de Diagnóstico Médico of Guadalajara’s Grupo Río Center (Jalisco, México). A T1-weighted turbo field echo (TFE) structural image was obtained for each subject with a 3-dimensional protocol (repetition time [TR] = 2.8 ms, echo time [TE] = 6.3 ms, 170 slices, and field of view [FOV] = 240 × 240 × 170). The scan time of the T1 had a duration of 2 minutes and 52 seconds per participant. The image acquisition was in the sagittal plane. For the functional images, a T2*-weighted (BOLD) image was obtained (TR = 2 ms, TE = 30 ms, FOV = 230 × 230 × 160, voxel size = 2.4 × 2.4 × 4 mm, 29 slices). The image acquisition was in the transverse plane. The resting-state scanning had a duration of 10 minutes. During scanning, the participants were instructed to relax, remain awake, and keep their eyes open and fixed on a cross symbol on the screen.

### 2.5. Image Preprocessing

The structural image data were analyzed using an FSL (FMRIB Software Library v5.0) preprocessing pipeline adapted under authorization from [61], with its parameters adjusted to fit our experimental data, including a motion correction procedure to solve the undesired head movements in the fMRI sessions. T1 images were reoriented to match the same axes as the templates, and a resampled AC-PC aligned image with 6 degrees of freedom (DOF) was created. All nonbrain tissue was removed to obtain an anatomic brain mask that would be used to parcel and segment the T1 data images. The final step involved registering our structural data images to the normalized space using the Montreal Neurological Institute reference brain based on the Talairach and Tournoux coordinate system [62].

### 2.6. Regions of Interest

The automated anatomical labeling (AAL) atlas [63] was used to define the regions of interest (ROIs). This atlas contains 45 cortical and subcortical areas in each hemisphere (90 areas in total), which are alternatively interspersed (available by request). To acquire the full signal of a given ROI, it is necessary to compute an average over the entire time-series of all the voxels of a given brain area following the AAL atlas.

In relation to the objective of the present study of distinguishing the brain connectivity patterns of MCI and healthy populations, we identified three of the principal RSNs: visual network, somatosensory system and DMN. DMN regions were divided into anterior, ventral and posterior subnetworks based on the classification proposed by [64]. The anterior DMN subnetwork included the anterior cingulate, paracingulate gyrus, insular cortex, and frontal and temporal poles. The ventral DMN subnetwork included the precuneus and middle cingulate, hippocampus and parahippocampal gyrus. The posterior DMN subnetwork included the posterior cingulate and precuneus, lateral parietal and middle temporal gyrus.

### 2.7. Statistical Analysis

To describe the clinical characteristics of the participants in the MCI and control groups, the median and interquartile range (IQR) of the score on every neuropsychological test was calculated. A Mann-Whitney *U* test was performed to compare these variables between the two groups to determine the groups’ homogeneity. The nonparametric Mann-Whitney test was chosen because of its suitability with small samples, as in the present study.

### 2.8. Functional Connectivity

To compare connectivity networks between ROIs between groups (MCI and Healthy), a Pearson correlation matrix was obtained for each group. We used the Pearson correlation coefficient for the subjects of the two groups over all of their time points. Thus, the correlation matrix had a dimension of 90 × 90, representing all the ROIs of the atlas.

To show the functional connectivity patterns in MCI patients and control participants, we conducted a cluster analysis for each group to classify all the ROIs of the atlas. Cluster analysis has been used before in the study of connectivity models [65]. For each group, we performed a hierarchical cluster analysis based on the Euclidean distance matrix obtained from the functional connectivity matrix.

Dendrograms for the two groups were created to optimize the visualization of these results.

To validate the optimal number of clusters, we calculated Dunn’s index [66]. This technique consists of verifying that the cluster groups are compact and well separated. For every cluster partition, *X_i_* represents the last cluster of every partition. Dunn’s index is defined as follows:

(1) $$DI=\frac{\begin{array}{c} min\\ 1\leq i\leq nc \end{array}\left\{\begin{array}{c} min\\ 1\leq j\leq nc,\;i\neq j \end{array}\;\left\{dist\;\left(X_{i},\;X_{j}\right)\right\}\right\}}{\begin{array}{c} max\\ 1\leq k\leq nc \end{array}\left\{diam\;\left(X_{k}\right)\right\}}\;\;\;$$

where *n_c_*= number of clusters, *dist* (*X_i_*, *X_j_*) = distance between two clusters, and *diam* (*X_k_*) = maximum distance between the elements of a *k* cluster.

In this case, the distance estimates between ROIs were established from Pearson’s linear correlation coefficients. The use of this association indicator seems appropriate when a cluster is established between variables with a large range of values, but in this case, the estimates between ROIs could reach a ceiling effect, as it was a normed index. To avoid this effect, the distance estimates were made from the transformations of all the correlation coefficients from the following expression:

(2) $$D_{ij}^{2}={\left(X_{i}-X_{j}\right)}^{\prime }S^{-1}\left(X_{i}-X_{j}\right)$$

where *S* is the variance-covariance matrix between the observed distributions of the ROIs. This transformation is based on Euclidean distance estimations used in those cases where the range of values of the distributions observed tends to be small. This is the case for the distributions of an fMRI signal that presents small variances. This transformation does not modify the range of the values of the coefficients that make up the matrices to analyze (0–1), but they are damped so as not to be affected by a small variability. This type of strategy has been applied in different backgrounds [67]. Finally, we conducted a study of functional segregation in the DMN. To study functional segregation, it was assumed that brain regions could develop specialized tasks by themselves, integrating all the information into more complex processing phases [68]. The interconnection of brain regions makes this possible because these regions form groups and clusters in the already known functional networks. One way to measure this segregation is the clustering coefficient, which can be calculated by computing the fraction of triangles around a given node of the network. These triangles can also be called 3-cycles and represent the nearest functional neighbors of an ROI that are functional neighbors of each other [67].

The correlation matrices used in the present study can also be used to infer the intensity of the signal correlations of the ROIs, considering that anatomical information from tractography was not available for the participants under study. Furthermore, the correlation coefficients between each two brain regions can be used as a proxy between those two ROIs. If we take into account a system with three ROIs, we define a simple structure of triangle, using the correlation coefficients between ROIs as the edges. For each triangle so defined, we calculate the areas through Heron’s formula:

(3) $$A=\sqrt{s\left(s-a\right)\left(s-b\right)\left(s-c\right)}$$

where a, b and c are the side lengths of the triangle and s is the semiperimeter, which is defined as follows:

(4) $$s=\frac{a+b+c}{2}$$

A threshold was applied to the 3-cycles to obtain only those areas whose sides were above 0.6 to obtain the most representative figure.

To perform the different analyses that were conducted in the present study, we used IBM SPSS Statistics 23, MATLAB and R software.

## 3. Results

### 3.1. Sociodemographic and Neuropsychological Characteristics

The analyses of the study included 7 men and 3 women in both groups. In the control group, the mean age was 56.1 years (sd = 10), and the mean education years were 14.3 (sd = 4.164). In the MCI group, the mean age was 61.7 years (sd = 7.424), and the mean education years were 13 (sd = 5.537). The median score and IQR for each neuropsychological test are provided in Table 1.

The Mann-Whitney *U* test was performed to determine if there were statistically significant differences between groups in these variables. The scores for daily normal activities (PAQ), geriatric depression (GDS), and memory and attention (NEUROPSI, PRMQ) showed statistically significant differences between the control and MCI participants. These results are consistent with the expected results because MCI participants had lower scores in cognitive tests in comparison to healthy controls. There were no differences in language (BNT) or general functioning (MMSE) between the groups.

Although statistically significant differences in geriatric depression scores were found, not a single participant could have been classified as depressed in either group. This is important because a depression diagnosis could influence the performance of the participants and their fMRI images.

### 3.2. Functional Connectivity

**Figure 1:**
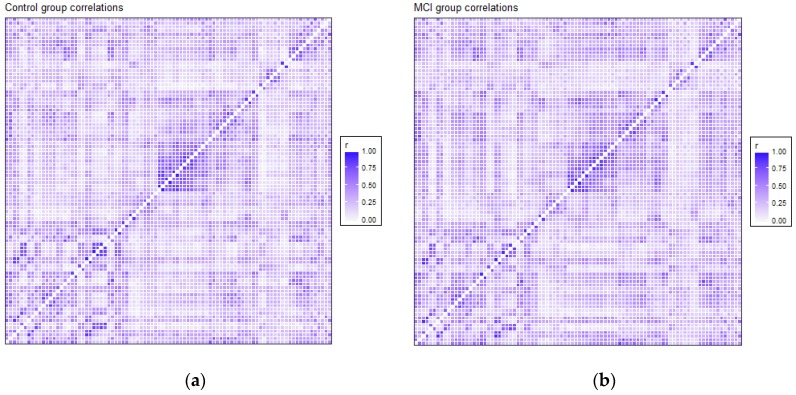
Correlation matrix for the control group (**a**) and MCI group (**b**). Every matrix includes the average of connectivity matrices of all participants in each group. The X and Y edges are formed by the 90 ROIs of the atlas.

**Figure 2:**
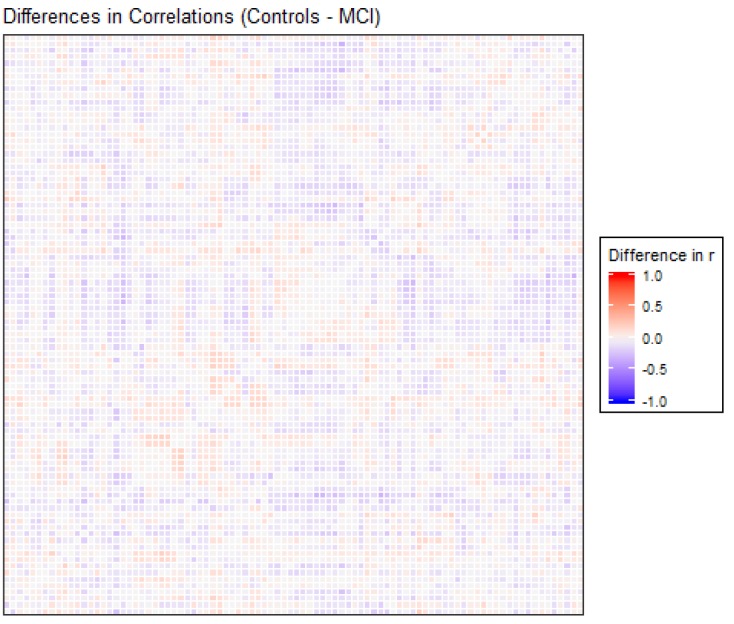
Differences in correlation coefficients between the control and MCI groups, as the result of the Control correlations matrix minus the MCI correlations matrix, are shown in Figure 1. The X and Y edges are formed by the 90 ROIs of the atlas.

The correlation matrices for the two groups in the study were very similar. Each of the elements of each matrix was estimated by means of the values observed for each pair of ROIs in each of the two samples separately. We opted for a robust value as the medium, given the reduced sample size. Anticorrelations were deleted to interpret only positive correlations between ROIs, and they were very scarce (approx. 6%). Figure 1 shows the intensity of the correlations between the AAL brain areas for the control and MCI groups. Many structures can be detected in the two matrices, but with different intensities between them, which will be detailed afterwards. A visual analysis of these matrices could not be performed because of their similarity. For that reason, we extracted the correlations that were different between the two matrices, and we selected only the differences that were greater than 0.2 to eliminate the least significant correlations, which could increase the presence of noise or interference. This criterion of significance was considered in view of the degrees of freedom and by estimating the minimum significant difference between two Pearson correlation coefficients through the use of Fisher parametric distributions. Figure 2 shows the difference matrix between the control and MCI groups.

Structures that showed more intensity in correlation coefficients, as shown in Figure 1, were related principally to the DMN, visual network and somatosensory system. These regions are usually activated in resting-state paradigms, as was the case in the present study.

There were 51 positive differences and 69 negative differences between the two correlation matrices. The negative differences principally involved the right supramarginal gyrus, left and right superior parietal regions, middle and inferior occipital areas, and especially the left and right temporal regions. The positive differences were more varied and involved many different regions, including the left and right inferior temporal and superior pole, left amygdala, right frontal regions and left and right hippocampus and parahippocampal gyrus. In Figure 2, the matrix of correlation differences between the groups is represented. Most of the differences in correlation coefficients were between 0.2 and 0.3 (no difference was greater than 0.4).

In order to clarify the content of the previous matrix of differences between correlations, we selected the differences (positive and negative) that had the greatest absolute values. The correspondence of each number with each brain ROI can be found in Table A1. The names of the ROIs involved are listed in Table A2.

**Figure 3:**
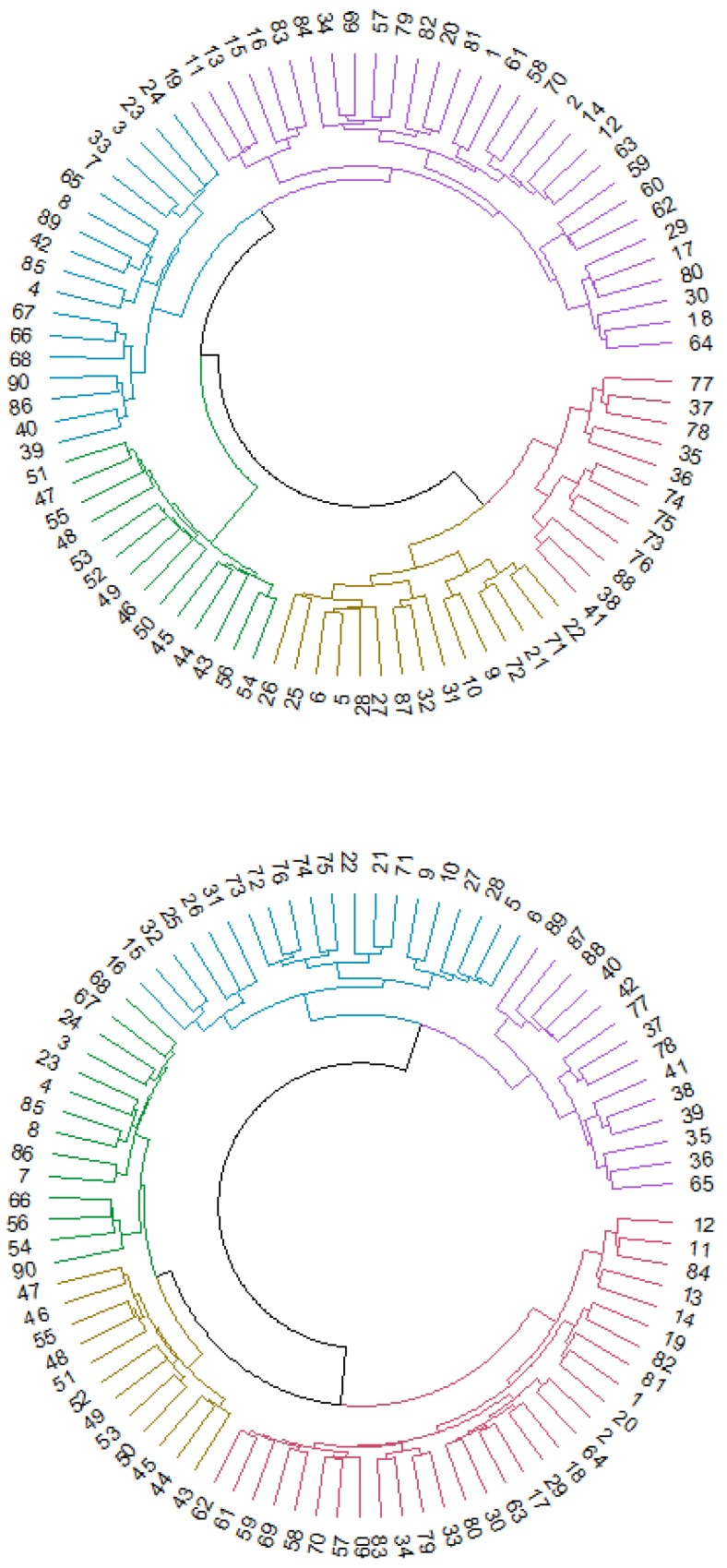
Cluster analysis for control (first dendrogram) and MCI (second dendrogram) participants. Every number represents an ROI of the AAL atlas.

Cluster analysis provided a classification of the 90 ROIs depending on the relationship and similarity of the regions. Figure 3 shows the dendrograms obtained from this analysis based on hierarchical clustering in Euclidean distance. Dunn’s index (DI) had a low score (DI = 0.54426), indicating nonoptimal values for the cluster analysis, so the results should be interpreted as preliminary results that need a more in-depth study in future research.

We obtained 5 clusters of regions for both dendrograms, but the distribution of the regions among the different clusters showed some differences when comparing the two dendrograms. In the control group, DMN regions were divided into different clusters represented by blue, purple, yellow and red. The blue cluster was composed basically of ventral and posterior DMN regions (left and right precuneus or left and right middle temporal gyrus, for example), and regions in the yellow cluster were principally anterior DMN ROIs (left and right anterior cingulum, for example). In the MCI dendrogram, blue, purple, green and red clusters contained DMN regions. Anterior, ventral and posterior DMN regions were divided into different clusters, in contrast to the first dendrogram. The sensorimotor network in the control group was assembled in the purple cluster, except for some regions that were classified in the yellow cluster. In the MCI group, most of the sensorimotor network regions were in the green group, and only a few were in the red cluster. Finally, the visual network system had mainly the same distribution in both dendrograms (represented by green in the control group and yellow in the MCI group).

Thus, the visual network did not show differences between groups, but the other RSNs seemed to be classified into different categories. Specifically, anterior DMN regions seemed to be the subnetwork with more differences, considering that they were assembled in a unique cluster in the control group but divided into different clusters in the MCI group. Additionally, the other regions that conformed to the DMN (ventral and posterior) changed classification, becoming more dispersed in the MCI dendrogram.

The regions of the triangles whose edges were represented by correlation coefficients were plotted to obtain their frequency distribution and to highlight any difference between the two groups. By visual inspection, the two groups showed a similar tendency in their area distributions, with more triangles of smaller areas and fewer triangles with larger areas. The *χ*^2^ test for given probabilities showed that the number of triangles was homogeneous in the two groups (*χ*^2^ = 2.214, *df* = 1, *p* = 0.1367). Then, a further analysis of the triangles’ areas was performed taking into account the differences in the frequency.

**Figure 4:**
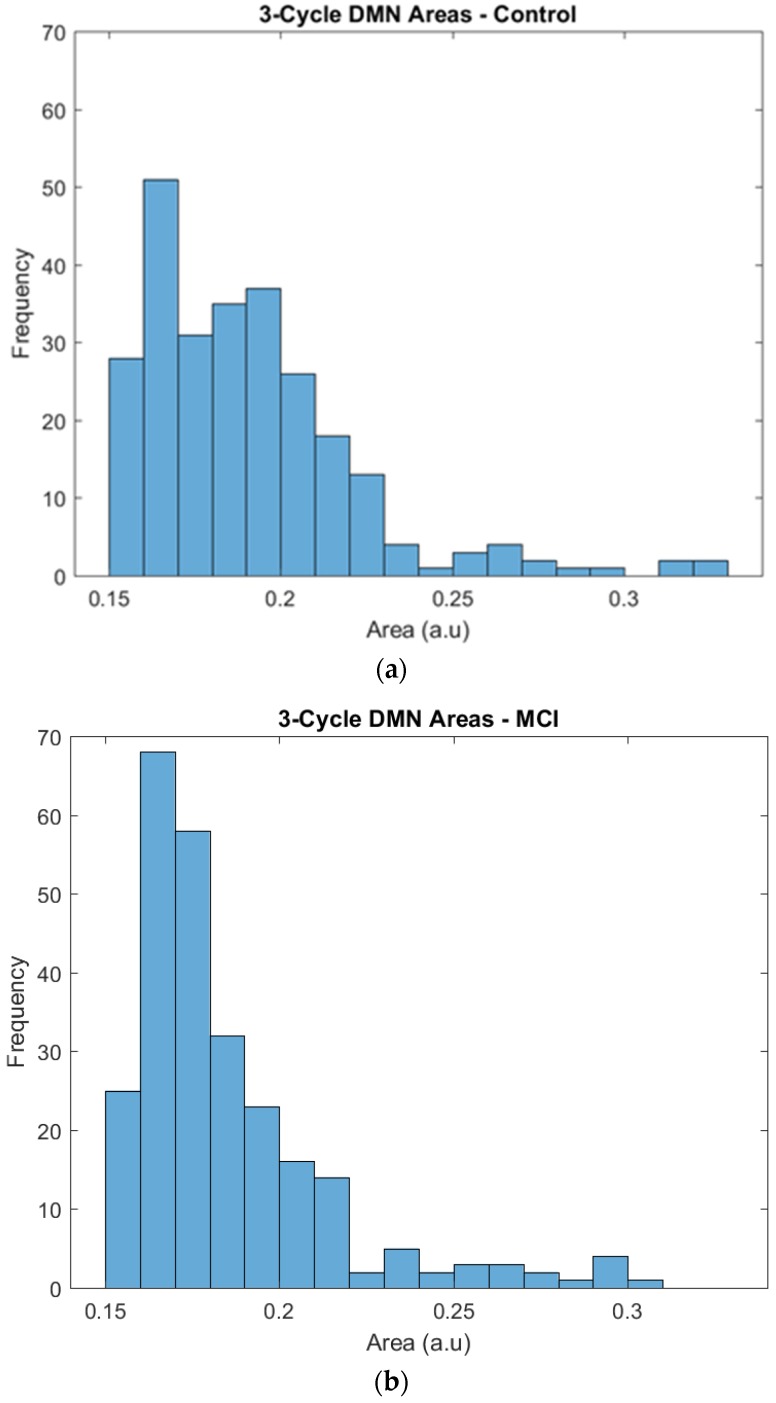
Frequencies of the 3-cycle areas in the control (**a**) and MCI (**b**) groups.

Specifically, the distribution of the larger surfaces was almost the same, but we found some differences in the distribution of the small areas that needed a deeper inspection. The control group showed a relatively constant number of triangles across the areas, and 0.15 and 0.2 were the most frequent areas. The frequencies of triangles with these areas ranged from 25 to 40, and the most frequent area was 0.16, with 50 triangles. The MCI group also showed a frequency peak at areas of 0.16 and 0.17, but it was particularly noticeable in comparison to the control group, as it had approximately 70 triangles in this peak, while the rest of the frequencies ranged from 20 to 35 (see Figure 4).

As described in previous results, there were differences in functional connectivity and segregation between the two groups, and although these differences were subtle, they were relatively noticeable in the details of the different analyses.

## 4. Discussion

The aim of the present study was to investigate the differences in functional connectivity between MCI patients and healthy individuals who were the same age, had the same level of education and were the same sex.

The results extracted from correlation matrices showed how resting-state connectivity patterns of the participants in both the MCI and control groups were similar to what was expected in these populations. Regions that showed more intensity in their connections were located in the DMN areas, as well as other usual RSNs, such as visual and sensorimotor networks. Although the structures of activation were mostly the same in both groups, the control group showed higher levels of intensity in DMN areas based on the correlation matrix, while the MCI participants had more activation in other regions.

Studying the classification of the 90 ROIs in each group, a cluster analysis revealed 5 clusters. The cluster classification of the two groups was notably different. Usually, the visual classification through dendrograms can help the study of these differences, but in this case, the inclusion of 90 ROIs that made this analysis more difficult because of the large number of regions included. However, examining the details of the obtained clusters in each group, the control group presented clusters that were more aligned with the natural classification of the different RSNs than the MCI group, whose individual clusters contained regions from almost every RSN. As an exception, the visual network was nearly stable in both groups, and thus, this network became the RSN that seemed to be least affected by the differences between MCI and healthy adults. 

In particular, the anterior DMN regions showed the largest differences between the groups. In the control group, most of the regions of the anterior DMN were classified in one cluster, while in the MCI group, these regions were dispersed across almost all the different clusters. These results indicated a more disperse connectivity in MCI patients and a less robust classification in this group.

On the cluster analysis, it is important to bear in mind that the value of the Dunn index indicated nonoptimal values in this analysis, so the results should be taken as preliminary. It will be important to expand the investigation by this method to be able to corroborate the present results.

Finally, the functional segregation analysis with the triangles complemented the information obtained from the previous analysis. The frequency distributions were, based on an initial visual inspection, relatively similar. Nevertheless, after a more detailed inspection, it was determined that the control group showed less variability between the different areas, with similar frequencies in the lower and higher parts of the distribution, and there was a relatively uniform progression. The MCI group showed a higher concentration of triangles in particular areas, especially in the 0.16 and 0.17 areas, and an abrupt change in the progression.

Previous studies have shown similar results related to some of our findings. The differences in the correlation matrix showed that, in effect, there existed different correlation coefficients between the two groups, in both the positive and negative directions, in similar proportions. When the values with magnitudes less than 0.2 were filtered, it was observed that most of the values were between 0.2 and 0.3 (both positive and negative), with no differences in the correlations greater than 0.4. These results confirmed that the differences in correlations were of medium-low intensity, and these results show that the differences between groups were subtle.

The regions involved in these differences presented some variations depending on whether the sign was positive or negative. Specifically, positive differences were found in regions involving sensorial integration, basic cognitive functions, the primary motor cortex and somatosensory cortex in relation to the visual network, the primary somatosensory cortex and the DMN. On the other hand, negative differences were detected in regions involving language processing, the visual network and the DMN in relation to the visual network, the primary auditory cortex and the DMN. The detection of changes in these regions was not surprising in a resting-state paradigm, but the positive and negative differences between these populations permitted a better understanding of the functioning of the different networks in each group.

Based on these considerations, the results suggest two potential interpretations. First, the decrease in functional connectivity in particular regions in MCI patients has been described in multiple articles [17,23,24,25,26]. Second, the increases in activation, which could be interpreted as a compensatory mechanism, have been previously found in MCI participants [16,17,18].

In addition, some of the findings of this study are similar to previous literature of this topic. In particular, Binnewijzend et al. found lower DMN connectivity in MCI individuals than in healthy controls, although the group with the greatest decrease was the AD group [27]. The results of [31] were in the same direction, finding significantly lower functional connectivity in the DMN of MCI participants than in the DMN of the control group. Recently, [28] confirmed decreased connectivity between the DMN and the other RSNs, such as visual and sensorimotor networks in MCI patients.

In our study of functional segregation by 3-cycle area, participants in the control group showed a uniform distribution of areas with a steadily decreasing progression; in contrast, the participants in the MCI group showed a marked concentration in the low part of the distribution, where the small triangle areas were located. This type of change in functional segregation and the relation between networks could be similar to results reported by [32], who showed changes in the modulation of the dorsal salience network in relation to the DMN when comparing MCI participants and young adults. The changes described in the distribution of triangles could be a response to the need of the participants with MCI to face the normal demands of brain functioning, in which case the activation of more triangles could help accomplish this goal. The control group showed a greater number of total triangles, but without any marked concentration of triangles in a particular area. Additional studies are required to obtain a deeper understanding of the details and possible explanations derived from these results.

Taking into account the present results related to correlation connectivity matrices and the triangle distribution in each group, it is interesting to examine the work of [69], who studied cortical functional connectivity in amnestic MCI patients. On the one hand, the results revealed a lower local DMN connectivity and a higher connectivity in the sensorimotor network. On the other hand, they detected lower connectivity patterns at a remote (not local) level in the DMN, as well as higher connectivity in the sensorimotor and attentional networks.

To the best of our knowledge, the results presented in this study in relation to the cluster analysis information have not been described in the published literature. Remarkable changes in cluster distributions could have been expected, given the differences in connectivity patterns that have been described widely before. However, the specific differences in the classification of the anterior DMN regions have not been previously described. In most of the investigations, the DMN network has been studied globally, although there have been articles in which the relation of a specific region of the DMN with another brain region was the goal of the study. Nevertheless, the division of the DMN into anterior, ventral and posterior regions, based on the proposal of [34], allowed a more fine-grained analysis in the present study. Therefore, future investigations using this classification could clarify the role of the anterior DMN regions in relation to the functional connectivity observed in MCI individuals compared to healthy adults with the same characteristics.

Several particularities and limitations of our study should be considered and resolved in future research. Limiting the correlations used to construct the triangles to those greater than 0.6 was done to include only the pairs of regions with a notable relation, avoiding those with low intensity. However, a more in-depth investigation is necessary to optimize the selection of this limit. The low number of participants in this study could seem a limitation of the investigation. Two years were needed to find 27 participants who met all the inclusion criteria, and 7 of them were eventually excluded because of excessive movement during the fMRI scan. However, the rigor of the inclusion and exclusion criteria represented our strong desire for a sample in which the probability of obtaining a false positive or a false negative was 0, and in fact, this need presupposes that a very exhaustive assessment is necessary, as will be discussed below. Another limitation related to the sample was the impossibility of establishing a separation into the different MCI subtypes. Participants in this group had no language impairment but presented alterations in memory and, in some cases, in attention. Most of the participants had amnestic MCI, and some of them had attentional deficits. Nevertheless, it was decided to create a single group including all MCI-diagnosed individuals, without incorporating these particularities, and to analyze the group as a whole to avoid the excessive fragmentation of a small sample that may cause major problems in the posterior analysis.

Furthermore, the use of triangles for the purpose of studying the connectivity characteristics between brain regions is still relatively new in this research area. It is true that the study of the activity between regions has been presented from a large number of different statistical approaches. However, the use of this specific technique of triangle areas has not been used in this population or to compare two different populations, as was done in the present research.

The inclusion and exclusion criteria of the participants in the present study are a key point that is important to highlight. These criteria were very restrictive, so it was more difficult than expected to find participants that perfectly fit in the MCI group as well as to find matched subjects for the control group. At the same time, these criteria guaranteed that the participants were perfectly classified and diagnosed. This guarantee provides much value to the present research, and the criteria did take into account the difficulties inherent to the MCI diagnosis. A group of clinical and neuropsychologists and a neurologist, experts in cognitive impairment, were responsible for the participant assessments, using a large number of tests related to the different neuropsychological domains. Thus, the rate of possible false positives in our sample was reduced, assuring that participants in the MCI group were appropriately diagnosed, and the participants in the control group had no alterations in any of the relevant cognitive domains.

## 5. Conclusions

In conclusion, the results of the present study corroborate the existence of differences in functional connectivity patterns in MCI individuals when compared to the patterns in cognitively preserved adults with the same characteristics (age, sex and education level). Most of the differences had been described and were in line with previous studies, especially the role of the DMN [27,31], which showed a decrease in connectivity but also increases depending on the region [17,28], and changes in the type of relation between regions in the different RSNs [32,69]. Finally, we detected a difference in the classification of the regions involved in the RSNs based on cluster analyses that revealed an exceptional effect in the regions of the anterior DMN, which showed more dispersion in classification in the MCI group than in the control group. Additional studies are needed to provide a deeper understanding of the characteristics of the anterior DMN and its role in this diagnosis and to confirm whether these differences might be a possible biomarker for MCI diagnosis.

## Figures and Tables

**Table 1:** Characteristics of the MCI and control samples.

Groups/Variables	PAQ	BNT	GDS	MMSE	NEUROPSI	PRMQ
	Median (IQR)	Median (IQR)	Median (IQR)	Median (IQR)	Median (IQR)	Median (IQR)
Control	0 (0)	58 (2)	1 (4)	27.5 (3)	111 (10)	26.5 (6)
MCI	1 (2)	57 (8)	5.5 (4)	27.5 (3)	95.5 (10)	39.5 (16)
Mann–Whitney-Wilcoxon *U* test (*p*-value)	23.5 (0.018) *	65.00 (0.251)	10.00 (0.0022) **	58.00 (0.537)	85.5 (0.0072) **	13.00 (0.0048) **

*p* < 0.05 (*) and *p* < 0.01 (**) are considered statistically significant.

## Appendix A

**Table A1:** ROI number and brain region correspondence from AAL Atlas.

ROI Number	Brain Region	ROI Number	Brain Region
1	Precentral_L	46	Cuneus_R
2	Precentral_R	47	Lingual_L
3	Frontal_Sup_L	48	Lingual_R
4	Frontal_Sup_R	49	Occipital_Sup_L
5	Frontal_Sup_Orb_L	50	Occipital_Sup_R
6	Frontal_Sup_Orb_R	51	Occipital_Mid_L
7	Frontal_Mid_L	52	Occipital_Mid_R
8	Frontal_Mid_R	53	Occipital_Inf_L
9	Frontal_Mid_Orb_L	54	Occipital_Inf_R
10	Frontal_Mid_Orb_R	55	Fusiform_L
11	Frontal_Inf_Oper_L	56	Fusiform_R
12	Frontal_Inf_Oper_R	57	Postcentral_L
13	Frontal_Inf_Tri_L	58	Postcentral_R
14	Frontal_Inf_Tri_R	59	Parietal_Sup_L
15	Frontal_Inf_Orb_L	60	Parietal_Sup_R
16	Frontal_Inf_Orb_R	61	Parietal_Inf_L
17	Rolandic_Oper_L	62	Parietal_Inf_R
18	Rolandic_Oper_R	63	SupraMarginal_L
19	Supp_Motor_Area_L	64	SupraMarginal_R
20	Supp_Motor_Area_R	65	Angular_L
21	Olfactory_L	66	Angular_R
22	Olfactory_R	67	Precuneus_L
23	Frontal_Sup_Medial_L	68	Precuneus_R
24	Frontal_Sup_Medial_R	69	Paracentral_Lobule_L
25	Frontal_Med_Orb_L	70	Paracentral_Lobule_R
26	Frontal_Med_Orb_R	71	Caudate_L
27	Rectus_L	72	Caudate_R
28	Rectus_R	73	Putamen_L
29	Insula_L	74	Putamen_R
30	Insula_R	75	Pallidum_L
31	Cingulum_Ant_L	76	Pallidum_R
32	Cingulum_Ant_R	77	Thalamus_L
33	Cingulum_Mid_L	78	Thalamus_R
34	Cingulum_Mid_R	79	Heschl_L
35	Cingulum_Post_L	80	Heschl_R
36	Cingulum_Post_R	81	Temporal_Sup_L
37	Hippocampus_L	82	Temporal_Sup_R
38	Hippocampus_R	83	Temporal_Pole_Sup_L
39	ParaHippocampal_L	84	Temporal_Pole_Sup_R
40	ParaHippocampal_R	85	Temporal_Mid_L
41	Amygdala_L	86	Temporal_Mid_R
42	Amygdala_R	87	Temporal_Pole_Mid_L
43	Calcarine_L	88	Temporal_Pole_Mid_R
44	Calcarine_R	89	Temporal_Inf_L
45	Cuneus_L	90	Temporal_Inf_R

**Table A2:** Pairs of ROIs with positive or negative differences between correlation coefficients of the two groups (in correspondence with Figure 2).

Positive Differences			Negative Differences		
First ROI	Second ROI	Difference	First ROI	Second ROI	Difference
8	10	0.3051	88	89	−0.332
41	84	0.2899	19	49	−0.3008
28	43	0.2894	19	59	−0.3006
37	84	0.2873	53	89	−0.2997
28	47	0.2728	19	50	−0.2994
28	45	0.2723	18	88	−0.2979
10	53	0.2701	13	60	−0.2783
78	83	0.2671	4	53	−0.2751
78	84	0.2568	56	64	−0.2666
31	41	0.253	53	85	−0.2664
10	55	0.252	8	88	−0.266
28	48	0.2514	14	88	−0.2598
10	89	0.2511	52	64	−0.2591
32	41	0.2494	19	52	−0.258
25	28	0.2417	18	40	−0.2577
16	70	0.2399	13	18	−0.2576
10	25	0.2386	18	26	−0.255
77	83	0.2362	1	18	−0.2536
76	79	0.2346	42	88	−0.2525
40	84	0.2341	14	26	−0.2507
28	41	0.2339	22	42	−0.2478
28	46	0.2327	18	32	−0.2471
1	65	0.2324	5	18	−0.2442
34	61	0.2299	60	88	−0.2436
33	41	0.2297	62	80	−0.2429
28	44	0.2275	46	85	−0.2417
4	10	0.2272	35	46	−0.2384
39	84	0.2269	19	60	−0.2376
33	39	0.2218	8	53	−0.2373
57	65	0.2211	18	59	−0.2368
34	41	0.2199	18	25	−0.2336
40	69	0.2185	13	80	−0.2313
33	90	0.2181	50	89	−0.2277
69	75	0.2172	58	59	−0.2266
1	34	0.2154	53	67	−0.2265
10	24	0.2154	56	62	−0.2254
37	57	0.2141	60	82	−0.2238
57	76	0.2139	59	80	−0.2218
41	75	0.2137	36	46	−0.2215
26	28	0.2132	2	88	−0.2207
33	37	0.2118	15	60	−0.2196
70	75	0.2115	50	85	−0.2177
10	87	0.2115	51	63	−0.2175
39	69	0.2113	67	80	−0.2173
34	90	0.2092	51	82	−0.2171
33	74	0.2089	13	52	−0.2157
69	76	0.2083	35	65	−0.2152
79	90	0.2076	19	46	−0.215
15	41	0.2063	13	88	−0.2134
33	40	0.2041	49	64	−0.213
11	65	0.2014	67	79	−0.2122
			26	82	−0.2122
			18	31	−0.2113
			50	64	−0.2113
			55	88	−0.2096
			51	64	−0.2092
			25	82	−0.2089
			46	77	−0.206
			52	63	−0.2053
			18	71	−0.2053
			18	22	−0.204
			38	78	−0.2036
			38	47	−0.2036
			18	51	−0.2025
			30	88	−0.2011
			19	53	−0.2004
			13	86	−0.2002
			64	86	−0.2001
			19	68	−0.2
